# Protein Interaction Mapping related to Becker Muscular Dystrophy

**Published:** 2019

**Authors:** Ali Azghar PEYVANDI, Farshad OKHOVATIAN, Majid REZAEI TAVIRANI, Mona ZAMANIAN AZODI, Mostafa REZAEI TAVIRANI

**Affiliations:** 1 Hearing Disorders Research Center, Shahid Beheshti University of Medical Sciences, Tehran, Iran; 2 Physiotherapy Research Centre, School of Rehabilitation, Shahid Beheshti University of Medical Sciences, Tehran, Iran; 3 Faculty of Medicine, Iran University of Medical Sciences, Tehran, Iran; 4 Proteomics Research Center, Shahid Beheshti University of Medical Sciences, Tehran, Iran; 5 Proteomics Research Center, Shahid Beheshti University of Medical Sciences, Tehran, Iran

**Keywords:** Becker muscular dystrophy (BMD), Protein-protein interaction, Map analysis, Gene ontology, Hub proteins

## Abstract

**Objective:**

Becker Muscular Dystrophy (BMD) is a neuromuscular disorder which is incurable. In this research protein interaction network of most associated proteins with BMD to provide better clarification of disorder underlying mechanism was investigated.

**Materials & Methods:**

The related genes to BMD were retrieved via string database and conducted by Cytoscape and the related algorithms. The network centrality analysis was performed based on degree, betweenness, closeness, and stress parameters. Gene ontology and clustering were performed via ClueGO analysis.

**Results:**

DMD as the super-hub as well as other central proteins including UTRN, TTN, DNM2, and RYR1 are important in BMD in terms of interactive features. The impairment of muscular contraction may be vital in BMD disease pathogenesis as it is the highlighted biological process term obtained by ClueGO analysis.

**Conclusion:**

DMD targeting may be the main concern for dystrophy clinical approaches. However, the other suggested proteins should be evaluated. Targeting these key proteins are required for treatment goals following extensive validation studies.

## Introduction

Becker Muscular Dystrophy is a less severe type of dystrophy than Duchenne muscular dystrophy (1). This type of neuromuscular disorder is mainly known with dysfunctional protein dystrophin (2). As there is no therapy available for BMD, molecular examination can be helpful in this regard (3). 

The potential molecules contributing to disease pathogenesis can be targeted for treatments (4, 5). Many investigations in this light have been conducted and provided further information about this disease and with possible usage in clinical goals (6). The analysis conducted up until today showed the contribution of many molecules for this disease that worth more evaluations (7, 8). However, due to the complex molecular nature of muscular dystrophy, more studies in terms of proteins as the functional level of cell is required (9). 

The way these proteins communicate can their linkage results in many different phenotypes are known as protein-protein interaction network (10). Any changes in this interaction unit may lead to phenotype alterations and sometimes the manifestation of a specific disease. This procedure is related to how the communication of these proteins can affect other proteins and the contributing pathways (11). Therefore, by analyzing these features the possible knowledge of molecular behavior of any kinds of diseases can be better understood (12). In addition, by examining proteins with prioritized topological features, potential nominates for biomarker discovery can be introduced. 

Moreover, more in-depth knowledge can be achieved through these studies by exploring the functional roles and biological processes related to these central proteins (13). On the other hand, expression changes in these types may result in vast dysregulation in the whole PPI network and accordingly, a disease phenotype may manifest (13). Etiology of Becker muscular dystrophy can be better explained by protein-protein interaction (PPI) network analysis and wide validation methods applications. Here, an introductory network value of this important muscular disease is suggested. 

## Materials & Methods

For the network construction, the used application was Cytoscape 3.4.0. (14). Through Cytoscape, STRING Database provided the essential information for interaction pattern by the use of different sources and combined confidence score (15). The setting for network construction was as follows: Number of nodes: 100 and combined confidence score cutoff= 0.5. However, only 93 one among the 100 genes were included in the constructed network.

 Following network construction, connectivity degree (D), betweenness, closeness, and stress as the key topological parameters were adopted to interfere the centrality properties of the constructed network. Nodes with high degree values are known as hub proteins. Hubs have many connections and any changes in them can result in extensive disruption of the whole interaction system. Tnode with high value of betweenness is called bottleneck node (16). For node evaluation, 10 top nodes based on the four mentioned centrality parameters have been selected. In this way, the central nodes of a network identify (17).

Further analysis was conducted based on gene ontology examinations. ClueGO+CluePedia Cytoscape plug-ins explored the gene ontology (GO) annotations (18, 19). Controlled vocabulary of three biological annotations including cellular component, molecular function, and biological process terms are implemented by GO source (20). The statistical test for this analysis was Kappa score. High kappa score indicates the higher possibility that terms group together. The cut off for here was set 0.5. In addition, genes per term was considered 3 and the percentage was set to 4. The minimum and maximum level of ontology were set to 3 to 8 as the default option, respectively. The *P*-value was also set to ≤0.05 and Bonferroni step down as the correction method. Two-sided (enrichment/depletion) based on hypergeometric test was also selected as the default option. 

## Results

**Figure 1:**
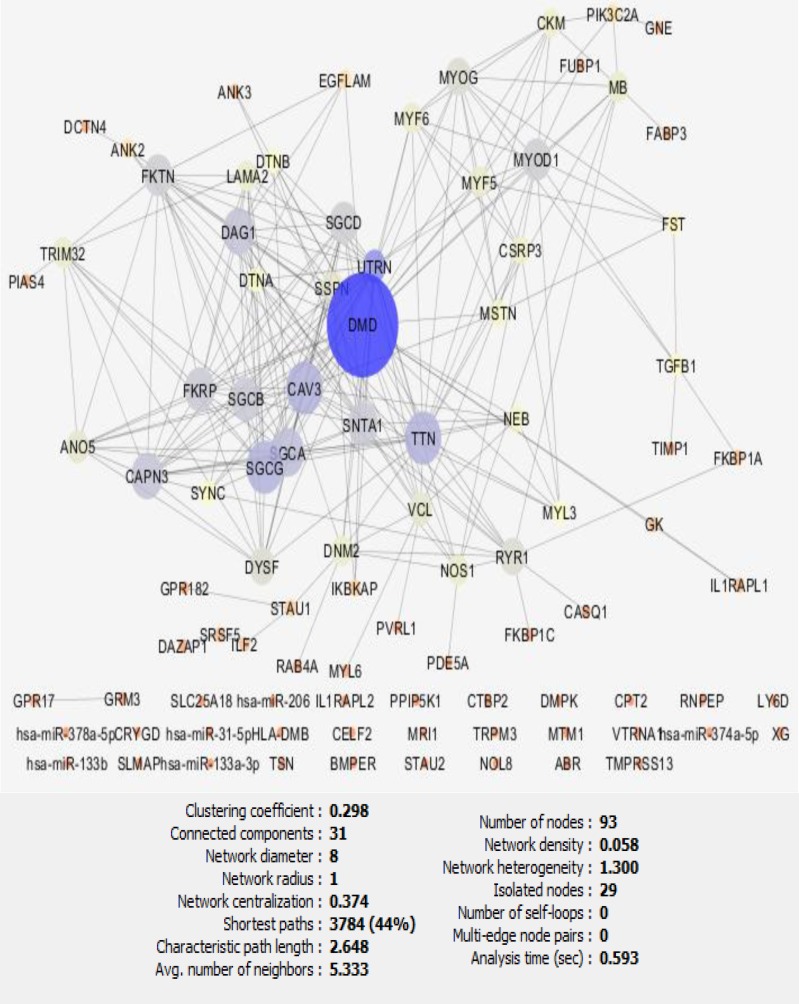
The network view of Becker muscular dystrophy analyzed by Cytoscape. Statistical properties of the network are shown in the down of the figure. Network consists of 93 nodes including 29 isolated nodes, one paired nodes and 62 organized nodes in the main connected component

Protein network construction of Becker muscular dystrophy via String Database and Cytoscape followed by is shown in Figure 1. 

**Figure 2:**
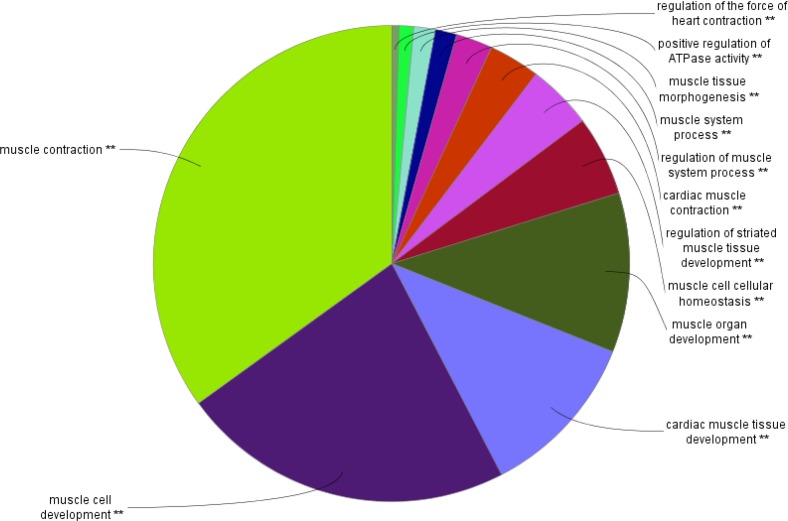
The pie chart shows significant contributed biological processes related to the main network of the Becker muscular dystrophy. The most highlighted group here is the green part. The network statistical properties are as follow: *P*<0.05, Kappa Score=0.5

Centrality analysis of the network was by the use of Network Analyzer. This algorithm is well established in Cytoscape and can provide essential topological parameters’ properties. Here, degree, betweenness, closeness, and stress as the most central characteristic of a PPI network are considered for centrality evaluation (Table 1). If a protein was hub or bottleneck and was included in the selected nodes based on closeness and stress was introduced as crucial proteins (Table 2). Gene ontology examination can be handled by the application of ClueGO. Here, the biological process evaluation of the main network was assessed (Figure 2).

**Table 1:** The 10 top nodes based on degree (D), betweenness (B), closeness (C) and stress (S)

**R**	**Gene Name**	**D**	**Gene Name**	**B**	**Gene Name**	**C**	**Gene Name**	**S**
1	DMD	39	DMD	0.406106	GPR17	1	DMD	4516
2	UTRN	25	DNM2	0.187341	GRM3	1	DNM2	2232
3	SGCG	19	STAU1	0.12623	DMD	0.663043	UTRN	1890
4	CAV3	19	RYR1	0.103262	UTRN	0.544643	TRIM32	1670
5	TTN	19	UTRN	0.095463	TTN	0.530435	STAU1	1462
6	SGCA	17	TTN	0.074896	CAV3	0.488	MYOD1	1318
7	CAPN3	16	MYOD1	0.074827	DAG1	0.484127	RYR1	1200
8	DAG1	16	VCL	0.073312	DNM2	0.480315	MYOG	966
9	SNTA1	15	ILF2	0.064481	RYR1	0.480315	VCL	928
10	FKRP	15	MB	0.043576	SNTA1	0.469231	ILF2	736

**Table 2:** The crucial nodes considering a hub or bottleneck gene included in the selected nodes based on closeness and stress

**R**	**Gene Name**	**Description**	**D**	**B**	**c**	**s**	**Disease score**
1	DMD	dystrophin	39	0.41	0.66	4516	4
2	UTRN	utrophin	25	0.10	0.54	1890	2.66
3	TTN	titin	19	0.07	0.53	-	1
4	DNM2	dynamin 2	9	0.19	0.48	2232	2.22
5	RYR1	ryanodine receptor 1 (skeletal)	12	0.10	0.48	1200	0.87

## Discussion

Topological examination implements remarkable properties of interaction basis of molecular concept of any kinds of diseases (21). Proteins with such characteristics are essential for network integrity and strength (22). In this study, proteins with such properties are suggested for BMD. In Figure 1, the first component of the network is a medium network that the most essential properties are contributing in this part. Other components are isolated or small communities not considered. No interactions between these nodes and the main network may be due to either undiscovered interactions or insignificant contribution of these proteins for BMD. The range of contribution of the isolated nodes to the disease is between 0.5-1 and for the other nodes is between 0.5-4 (Table 2).

DMD has the highest score which is 4. Nodes within main network are more associated with BMD than the other ones to the disease. DMD plays a role as the core of the network. Furthermore, DMD is the highest scored in terms of degree and betweenness values, which is a top-rated hub and bottleneck node (hub-bottleneck node). The association between this protein and BMD is strong as indicated as disease score. By considering the five crucial genes, in fact, 8.5% of the nodes were considered as key proteins. These nodes include DMD, UTRN, TTN, DNM2, and RYR1. These proteins were again searched against literature and finally, the result showed that they are highly pertinent to BMD. In addition to high values of centrality parameters, the disease score values of these central nodes are is the highest compared to other nodes.

Utrophin the other key protein is well known as a relevant protein to the two Duchenne and becker muscle dystrophies (23-25). Tight correlation between dystrophin and utrophin expression in Duchenne and becker patient is proved and discussed (26). Titin, the third key protein in the introduced possible biomarker panel is a giant sarcomeric filamentous polypeptide molecule. It is a component of striated muscle. There are evidence that titin plays a fundamental role in maintaining sarcomeric structural integrity. Responsibility for the passive elasticity of muscle is the main biological role of titin (27-29). 

The role of dynamin-2 (DNM2) in pathology of autosomal dominant centronuclear myopathy is investigated and reported by several researchers. This myopathy is usually a mild and clinically heterogeneous muscle disorder. Muscle weakness is the known characteristic of the patients. The nuclei are centralized in the biopsy of the patients (30-32).

Encoding isoform ryanodine receptor in the skeletal muscle by RYR1 gene is reported. This protein has a fundamental role in the excitation-contraction coupling process in the skeletal muscle. Control of calcium homeostasis by RyR1 is a significant role of this gene because intracellular calcium channel has a crucial role in muscle contraction (33, 34).

Centrality analysis indicates that while DMD has key properties in the network, other associated proteins may be fundamental in the BMD. Apparently, DMD is well-documented in Becker muscular dystrophy as well (35-37). Muscular dystrophy may trigger from malfunction of this protein. Role and centrality of this protein to be assessed also in other muscular dystrophies. 

Gene ontology analysis can express different properties of a group of proteins. The proteins contributing in a same network of BMD are inspected for biological process analysis. The biological process examination of BMD related proteins with different scores show that the Muscular Contraction is the most important biological process for BMD pathogenesis. Other processes are also important in this disease. Therefore, dysregulation of the assigned BMD proteins may lead to disruption of biological processes and mainly the highlighted one namely, Muscular Contraction. Muscle cell development and cardiac muscle tissue development are introduced as the second and the third important biological processes. As it is depicted in Figure 2, about 10 processes that tightly are related to the control of muscle function are listed.

**In conclusion, **DMD targeting may be the main concern for dystrophy clinical approaches. However, the presence of the other central proteins in the introduced panel also is confirmed by literature. Validity of this finding is assessed via more complementary investigation. 
